# Association between sleep quality and time with energy metabolism in sedentary adults

**DOI:** 10.1038/s41598-020-61493-2

**Published:** 2020-03-12

**Authors:** Lucas Jurado-Fasoli, Sol Mochon-Benguigui, Manuel J. Castillo, Francisco J. Amaro-Gahete

**Affiliations:** 1 Department of Medical Physiology, School of Medicine, University of Granada, 18016 Granada, Spain; 2 PROmoting FITness and Health through physical activity research group (PROFITH), Department of Physical Education and Sports, Faculty of Sport Sciences, University of Granada, Granada, Spain; 3 Camilo José Cela University, Madrid, Spain

**Keywords:** Metabolic syndrome, Nutrition

## Abstract

The aim of the present study was to investigate the relationship of sleep quality and time with basal metabolic rate (BMR) and fuel oxidation in basal conditions and during exercise in sedentary middle-aged adults. We also studied the mediation role of dietary intake and adherence to the traditional Mediterranean Diet in the relationship between sleep parameters and energy metabolism parameters.A secondary analysis of the FIT-AGEING study was undertaken. 70 middle-aged sedentary adults (40–65 years old) participated in the present study. Sleep quality was assessed using the Pittsburgh Sleep Quality Index (PSQI) and wrist accelerometers (ActiSleep, Actigraph, Pensacola, Florida, USA) for 7 consecutive days. BMR was measured with indirect calorimetry and fuel oxidation was estimated through stoichiometric equations. Maximal fat oxidation was determined by a walking graded exercise test and dietary intake with 24 h recalls. Adherence to the traditional Mediterranean diet was assessed through the PREDIMED questionnaire. PSQI global score (poor sleep quality) was associated with lower basal fat oxidation (BFox), both expressed in g/min and as a percentage of BMR, independently of confounders. We did not find any association between other sleep and energy metabolism parameters. No mediating role of the dietary intake or PREDIMED global score was observed in the association of PSQI and BFox. In conclusion, our study showed that a subjective poor sleep quality was associated with lower BFox, which is not mediated by dietary intake in sedentary adults.

## Introduction

Cardiometabolic diseases and obesity are the leading causes of death in developed countries, becoming an epidemic in the last years^1,2^. Unhealthy diets represent one of the top risk factors for cardiometabolic diseases and obesity, developing a positive energy balance^3^. Simultaneously, a low basal metabolic rate (BMR), an impaired meal-induced thermogenesis and low physical activity levels could result in a reduced total energy expenditure^4^. This low total energy expenditure coupled to high energy intake could produce a gradual weight gain and visceral adipose tissue deposition, increasing the risk of cardiometabolic diseases and obesity^5^.

The ability to oxidize fat as a fuel is considered an important metabolic health parameter^6^. An impaired ability to oxidize fat is associated with an increased risk of obesity, type 2 diabetes mellitus, cardiovascular disease, metabolic syndrome, cancer and systemic inflammation^7^. Therefore, fat oxidation in basal conditions (BFox) and maximal fat oxidation during exercise (MFO) are considered markers of metabolic health^8–10^.

Sleep pattern variations, including a decrease in the quality and quantity of sleep, have been shown to be also a risk factor for the development of obesity and cardiometabolic diseases^11^. These changes in sleep quality and quantity disrupt the circadian rhythms and may have deleterious consequences on people health^12^. Previous studies have provided a causal link between short sleep duration and poor sleep quality with pathological metabolic consequences due to the disruption in the circadian rhythms and increasing levels of adiposity^11,13^. Metabolic regulation is not an output function of the circadian system^12^. However, nutrient, energy and redox levels signal back to cellular clocks to reinforce circadian rhythms and to adapt physiology (i.e. hormones, body temperature, nervous system) to temporal tissue-specific requirements^11,13^. In this sense, previous studies have demonstrated that poor sleep quality and quantity may decrease BMR and BFox^14^, and sleep deprivation may not affect MFO in young adults^15^. However, there are no studies testing the associations between sleep quality and time (both subjective and objective) with BMR and fuel oxidation in basal conditions and during exercise.

One of the possible causes of the relationship between sleep parameters with BMR and fuel oxidation could be the dietary modifications. In this sense, unhealthy sleep patterns could increase food consumption and consequently energy intake through several previously-explained potential mechanisms^16^. Previous studies have shown that dietary intake could influence BFox and MFO^8,17,18^. Concretely, a high-fat, low-carbohydrate intake could increase BFox and MFO^6,19^. As well as, the lack of sleep can increase the consumption of high fat energy-dense foods^16^, which theoretically may modify BFox and MFO. However, there is a lack of evidence investigating the mediating role of dietary intake in the relationship of sleep outcomes with energy metabolism parameters.

Therefore, the aim of the present study was to investigate the relationship of subjective and objective sleep quality and time with BMR and fuel oxidation in basal conditions and during exercise in sedentary middle-aged adults. We also aimed to study the mediation role of dietary intake and adherence to the traditional Mediterranean Diet between sleep parameters and energy metabolism parameters in sedentary middle-aged adults. The present study undertakes a secondary analysis of the FIT-AGEING study.

## Materials and Methods

### Participants and design

A total of 70 (36 women) middle-aged sedentary adults (40–65 years old) participated in this cross-sectional study. The participants were enrolled in the FIT-AGEING study^20^, an exercise-based randomized controlled trial (clinicaltrial.gov: ID: NCT03334357). Data for these subjects were collected at baseline data collection in the FIT-AGEING study. All of them declared: (i) to be non-physically active (<20 min of moderate-intensity physical activity on 3 days/week), (ii) to have a stable weight (weight changes <5 kg) over the last 5 months, (iii) to be healthy and (iv) to be free of medication (medication for thyroid, betablockers, benzodiazepines, glucose lowering medication, or cardiovascular medication) during the last 5 months. All participants gave their oral and written informed consent before the beginning of the intervention. The study was approved by the Ethics Committee on Human Research of the University of Granada and the Andalusian Health Service (SAS) (CEI-Granada) (0838-N-2017). The study protocols and experimental design were applied according to the last revised ethical guidelines of the Declaration of Helsinki. All assessments were made at the Sport and Health University Research Institute (iMUDS, Granada, Spain) during September and October 2016 and September and October 2017.

### Anthropometric and body composition measurements

Anthropometric variables were measured by a certified anthropometrist [the International Society for the Advancement of Kinanthropometry (ISAK)] following the ISAK guidelines^21^. Both body weight and height were assessed using an electronic scale and stadiometer (Seca model 799, Hamburg, Germany), and the body mass index (BMI) was calculated as (weight [kg]/ height^2^ [m]). A dual-energy X-ray absorptiometry scanner (Discovery Wi, Hologic, Inc., Bedford, MA, USA) was used to measure body composition following the manufacturer’s recommendations. We conducted the quality controls, the positioning of the participants and the analyses of the results following the manufacturer’s recommendations. An automatic delineation of the anatomic regions was performed by the software APEX 4.0.2. We acquired spine phantom quality control scans on each study day. The results displayed lean mass and fat mass and the lean mass index (LMI) and the fat mass index (FMI), which were calculated as (lean mass [kg]/ heigh^t2^ [m]) and (fat mass [kg]/ height^2^ [m]) respectively.

### Sleep quality and time assessment

The Pittsburgh Sleep Quality Index (PSQI) is a self-report tool which consists of 19-item scale that provides 7 component scores (ranges 0–3): (i) subjective sleep quality (very good to very bad), (ii) sleep latency (≤15 minutes to >60 minutes), (iii) sleep duration (≥7 hours to <5 hours), (iv) sleep efficiency (≥85% to <65% hours sleep/hours in bed), (v) sleep disturbances (not during the past month to ≥ 3 times per week), (vi) use of sleeping medications (none to ≥ 3 times a week), and (vii) daytime dysfunction (not a problem to a very big problem), with a total global score ranging from 0 to 21^22^. A PSQI global score higher than 5 indicates poor sleep quality^22^.

Objective characteristics of sleep-wake cycles were monitored with a wrist-worn accelerometer (ActiSleep, Actigraph Pensacola, Florida, USA) for 7 consecutive days (24 hours/day)^20^. Participants received detailed information on how to wear the accelerometer and were asked to remove it only for water activities. It was also recorded the times in which the participants went to bed every night, woke up every morning and removed the device every day. The accelerometers used an epoch length of 5 seconds and a frequency rate of 100 Hz to store raw accelerations^23^. The raw accelerations were exported in “.csv” format using ActiLife v. 6.13.3 software (ActiGraph, Pensacola, FL, US) and processed using the GGIR package (v. 1.6–0, https://cran.r-project.org/web/packages/GGIR/index.html)^24^ in R (v. 3.1.2, https://www.cran.r-project.org/). We derived the Euclidean Norm Minus One G (ENMO) as √(x^2 + y^2 + z^2) −1G (where 1 G ~ 9.8 m/s2) with the accelerometer’s z angle to describe sleep patterns. We used a previously published algorithm combining data from the accelerometers and diary reports to detect sleep period time^25,26^. According to this algorithm, sleep was defined as any period of sustained inactivity, in which there were minimal changes in the arm angle (i.e., as much 5 degrees for 5 minutes periods) during a period recorded as sleep by the participant in their diary reports^25^. The following variables were analyzed: total sleep time (minutes slept between bedtime and wake time), sleep efficiency (percentage of time asleep while in bed) and wake after sleep onset (minutes awake between sleep onset and wake time). It is to note that only the participants wearing the accelerometers for ≥16 hours/day for at least 4 days (including at least 1 weekend day) were included in the final analyses (i.e. a total of 4 participants did not meet these conditions)^23^. The mean accelerometer wear-time for the 70 participants included in the final analyses were 6.7 days (4.3% of non-wear time).

### Basal metabolic rate and fuel oxidation in basal conditions

Subjects were told to arrive at the laboratory in fasting condition of at least 8 h in a motor vehicle and to avoid any moderate/vigorous physical activity in the previous 24 h/48 h respectively; all were required to confirm that they had met these conditions. The evening meal consumed by the subjects prior to fasting was previously standardized: an egg omelet with fried tomato and boiled rice.

BMR and fuel oxidation in basal conditions were measured through indirect calorimetry (IC) following the current scientific consensus^27^. All tests were conducted in the same quiet room with controlled room temperature (i.e. 22–24 °C) and humidity (i.e. 35–45%). IC measurements were performed during 30-minute periods with a CPX Ultima CardiO2 system (Medical Graphics Corp, St Paul, MN, USA) employing a neoprene face-mask with no external ventilation^27^.

The Ultima CardiO2 metabolic cart device assessed oxygen consumption (VO2) using a galvanic fuel cell, and carbon dioxide production (VCO2) via non-dispersive infrared analysis using a breath-by-breath system^28^. A gas calibration using 2 standard gas concentrations and a flow calibration using a 3-L calibration syringe were performed following the manufacturer’s recommendations. Prior to the start of the BMR assessment, the subjects reclined on a bed for ~30 min in a comfortable supine position covered by a sheet^29,30^. During the assessment, participants laid on a bed in a supine position and were instructed to breathe normally and not to talk, fidget or sleep.

The first 5 minutes of each measurement were discarded and the most stable 5-min period that met steady state criteria (i.e. coefficient of variation <10% in VO2, CO2, minute ventilation, and coefficient of variation <10% in respiratory exchange ratio) was considered for further analyses following previous studies^29–32^. The Weir’s abbreviated equation^33^ was used to estimate the BMR expressed in kcal/day and also calculated with respect to the lean mass (BMR_LM_). The Frayn’s equation was used to estimate BFox and basal carbohydrate oxidation (BCHox) expressed in g/min^34^. The BFox and BCHox were also expressed as a percentage of the BMR.

### Maximal fat oxidation during exercise assessment

MFO and the intensity that elicit MFO (FATmax) were assessed in a different day of the BMR and BFox/BCHox test (i.e. interval 3 to 15 days). Participants were asked to arrive at the laboratory in a fasted state of 6 hours and to avoid any physical activity both moderate (24 h) and vigorous intensity (48 h) before the measurement.

A walking graded test on a treadmill (H/P/cosmos pulsar, H/P/cosmos sports & medical GmbH, Nussdorf-Traunstein, Germany) was performed to calculate MFO and FATmax following a previously validated methodology^35^. Briefly, the protocol started assessing the maximal walking speed of each participant^35–37^. After ~3 minutes resting, the walking graded test started with a 3-minute warm up at 3.5 km/h. Subsequently, the treadmill speed was increased 1 km/h every 3 minutes until the maximal walking speed was reached. Thereafter, the treadmill gradient was increased 2% every 3 minutes until the respiratory exchange ratio was above 1.0. An automated gas analysis system (CPX Ultima CardiO2; Medical Graphics Corp, St Paul, MN) was used to record breath-by-breath gas exchange measurements. Participants wore an oronasal mask (model 7400, Hans Rudolph Inc, Kansas City, MO, USA) equipped with a prevent™ metabolic flow sensor (Medgraphics Corp, Minnesota, USA). Gas analysis systems were calibrated following the manufacturer’s recommendations. VO2 and VCO2 were averaged over and the last 60 seconds of each graded exercise protocol stage. Frayn’s equation was used to estimate fat oxidation rates^34^. These fat oxidation values were plotted against the relative-exercise intensity, expressed as the percentage of maximum oxygen uptake (VO2max); a third-degree polynomial curve was built to determine MFO and FATmax^38^. MFO was also expressed as MFO_LM_ in order to relativize it to the lean mass. Maximal carbohydrate oxidation was not included in the analyses since it is not a key factor of energy metabolism during exercise^39^. Indeed, our recent systematic review has analyzed a total of 112 studies which included data about fuel oxidation during exercise^37^. None of those studies reported maximal carbohydrate oxidation during exercise.

### Cardiorespiratory fitness assessment

VO2max was determined using a maximum treadmill (H/P/Cosmos Pulsar treadmill, H/P/Cosmos Sport & Medical GMBH, Germany) exercise test following the modified Balke protocol, which has been extensively validated^40^. In short, the warm up consisted in walking at 3 km/h for 1 minute followed by 2 minutes at 4 km/h. The incremental protocol started at a speed of 5.3 km/h (0% grade), which was kept constant with the gradient increasing by 1% every minute until the participants reached their volitional exhaustion. We used the same indirect calorimetry and software as in the MFO assessment.

### Dietary intake assessment

Diet was assessed using three 24-hour recalls carried out on 3 separate days (2 weekdays and 1 weekend day) by a qualified and trained research dietitian. Dietary recalls were done on different days than the MFO and VO_2_ assessments.

In the face-to-face interviews, the participants were asked to recall all food consumed during the previous day. The interviews involved a detailed assessment and description of the food consumption using colored photographs of different-size portions of food to improve the participants’ accuracy of food quantification^41^. These data were introduced by two independent qualified and trained dietitians in the EvalFINUT® software. Energy, macronutrient, fiber, lipid profile and ethanol intake data were obtained by EvalFINUT®, which is based on the USDA (United States Department of Agriculture) and BEDCA (“Base de Datos Española de Composición de Alimentos”) databases.

Dietary energy density was calculated by dividing the energy contained in food and beverages (excluding water) by the total weight of daily food and beverages (expressed as kcal/g)^42^. Energy and weight data of daily food and beverages were obtained from the 24-hours recalls.

The traditional Mediterranean diet is associated with a lower prevalence of chronic diseases (i.e. obesity, metabolic syndrome, cardiovascular diseases, cancer) and mortality^43^. The adherence to the traditional Mediterranean Diet (MedDiet) was estimated by using the 14-point questionnaire of adherence to the MedDiet used and validated in the PREDIMED trial^44^. The PREDIMED questionnaire includes 12 questions related to frequency intake of key foods and 2 questions related to specific dietary habits of the MedDiet. Each question scores 0 or 1 point. The global score ranges from 0 to 14, being 0 points null adherence and 14 points complete adherence to the MedDiet. The PREDIMED questionnaire proved to be very useful in a large Spanish cohort for a quick adherence estimation to the traditional MedDiet^44^.

### Statistical analysis

The sample size and power calculations were made based on the data of a pilot study of the FIT-AGEING study^20^. This study aimed to compare the influence of different exercise programs on BMR, BFox and MFO in sedentary middle-aged adults. We based the sample size calculations on a minimum predicted change in MFO of 0.05 g/min between the intervention groups and the control group, and an SD for this change of 0.05 g/min. A sample size of 17 participants was predicted to provide a statistical power of 80%, considering a type I error of 0.05. Assuming a maximum loss of 25% at follow-up, we decided to recruit at least 20 participants for each group (N = ~80 individuals). The present study is based on a secondary analysis using baseline data from the FIT-AGEING study, and therefore a specific sample size calculation was not conducted.

We used the Shapiro–Wilk test, visual check of histograms, Q-Q and box plots to verify all variable distributions. The descriptive parameters were reported as mean and standard deviation. Given that we did not observe a sex interaction, we conducted the analysis including men and women together. Simple linear regressions were performed to examine the association between sleep time and quality (PSQI global score, total sleep time, sleep efficiency and wake after sleep onset) with BMR, BMR_LM_, BFox, BCHox, MFO, MFO_LM_ and FATmax. We also conducted multiple linear regression models to test these associations after adjusting for sex (Model 1), sex and age (Model 2) and sex, age and FMI (Model 3).

Pearson correlation was performed to assess the association between sleep parameters and dietary outcomes. Effect modification analyses were conducted to test the joint effects of dietary intake (dietary intake outcome*sleep outcome) and sleep quality on energy metabolism. To quantify the mediating role of dietary intake (i.e. energy, macronutrient, fiber, ethanol and lipid profile intake, and PREDIMED total score) in the relationship between sleep parameters and BMR and fuel oxidation, we conducted mediation analyses^45^. We used the PROCESS macro version 3.3, model 4 with 5,000 bias-corrected bootstrap samples and 95% confidence intervals. Bootstrapping is a nonparametric resampling procedure that does not require the assumption of normality of the sampling distribution^46^ The mediation was estimated using the indirect effect, which indicates the change in the effect of the independent variable on the outcome that can be endorsed to the proposed mediator. Indirect effects (a × b paths) with confidence intervals not including zero are interpreted as statistically significant^47^ which could occur regardless of the significance of the total effect (c path, effect of the independent variable on the dependent variable) and the direct effect (c’ path, effect on the dependent variable when both the independent and the mediator variables are included as independent variables)^45^. To quantify how much of the total effect was due to the mediation, we calculated the percentage of mediation ([indirect effect / total effect] × 100) provided when the total effect was larger than the indirect effect with the same direction^45^.

All analyses were conducted using the Statistical Package for Social Sciences (SPSS, v. 25.0, IBM SPSS Statistics, IBM Corporation) and the level of significance was set at <0.05. Graphical presentations were prepared using GraphPad Prism 8 (GraphPad Software, San Diego, CA, USA).

### Ethical standards

Ethical approval for the study was given by the Ethics Committee on Human Research at the University of Granada and Servicio Andaluz de Salud (CEI-Granada) (0838-N-2017). Written informed consent was obtained from all subjects. This study was in accordance with the last revised ethical guidelines of the Declaration of Helsinki.

## Results

The characteristics of the study sample are shown in Table 1. We observed an inverse association between total sleep time and BMR (P < 0.001; Table 2), which disappeared after including sex, age and FMI in the model (all P > 0.105; Table 2). No association was found between the remaining subjective or objective sleep parameters with BMR and BMR_LM_ (all P > 0.071; Table 2), neither when we accounted for confounders.

**Table 1:** Descriptive parameters.

	All (N = 70)		Men (N = 34)		Women (N = 36)	
	Mean	SD	Mean	SD	Mean	SD
Age (years)	53.4	(4.9)	54.2	(5.2)	52.7	(4.7)
***Body composition parameters***						
Body mass index (kg/m^2^)	26.8	(3.8)	28.5	(3.5)	25.3	(3.4)
Lean mass (kg)	44.0	(11.7)	54.2	(6.3)	34.4	(5.7)
Lean mass index (kg/m^2^)	15.3	(2.9)	17.5	(2.0)	13.2	(1.8)
Fat mass (kg)	30.2	(8.4)	31.3	(9.5)	29.2	(7.3)
Fat mass index (kg/m^2^)	10.8	(3.1)	10.2	(3.2)	11.3	(3.0)
***Sleep quality parameters***						
PSQI global score	5.5	(3.5)	4.7	(3.2)	6.3	(3.7)
Total sleep time (min)	360.0	(48.9)	338.0	(46.3)	381.3	(41.8)
Sleep efficiency (%)	84.1	(11.9)	83.9	(7.5)	84.3	(14.9)
Wake after sleep onset (min)	62.2	(25.9)	65.8	(32.4)	58.7	(17.0)
***Basal metabolic rate and fuel oxidation in basal oxidation***						
BMR (kcal/day)	1511.3	(366.3)	1805.5	(244.8)	1233.5	(211.0)
BMR_LM_ (kcal/kg_leanmass_/day)	35.2	(7.3)	33.6	(5.3)	36.8	(8.5)
BFox (g/min)	0.05	(0.04)	0.06	(0.05)	0.04	(0.02)
BFox (% BMR)	45.0	(29.7)	45.6	(32.7)	44.4	(27.1)
BCHox (g/min)	0.11	(0.1)	0.14	(0.11)	0.10	(0.07)
BCHox (% BMR)	42.4	(31.5)	44.0	(34.7)	40.9	(28.6)
***Fuel oxidation during exercise***						
MFO (g/min)	0.29	(0.09)	0.35	(0.09)	0.24	(0.04)
MFOLM (g/kg_leanmass_/day)	6.8	(1.6)	6.4	(1.5)	7.1	(1.7)
Fatmax (% VO2max)	43.1	(10.5)	41.6	(10.3)	44.4	(10.7)
***Dietary intake***						
Energy (kcal/day)	2147.7	(739.8)	2206.8	(965.2)	2061.0	(459.0)
Dietary energy density (kcal/g/day)	1.2	(0.5)	1.3	(0.7)	1.1	(0.2)
Fat (g/day)	88.6	(25.4)	90.3	(26.1)	87.3	(25.4)
Protein (g/day)	90.0	(39.6)	92.5	(48.8)	85.9	(27.1)
Carbohydrate (g/day)	227.4	(120.0)	233.9	(158.8)	215.2	(61.6)
Fiber (g/day)	31.7	(28.5)	31.3	(26.1)	33.4	(32.6)
Ethanol (g/day)	9.9	(11.3)	11.3	(12.3)	8.2	(9.9)
SFA (g/day)	22.2	(7.4)	25.5	(6.7)	19.5	(7.0)
MUFA (g/day)	41.2	(13.9)	47.0	(12.7)	37.0	(13.3)
PUFA (g/day)	12.8	(5.0)	14.3	(5.3)	11.8	(4.5)
Cholesterol (mg/day)	279.2	(106.0)	295.9	(118.9)	261.7	(94.4)
PREDIMED total score	9.5	(1.9)	9.0	(2.0)	9.8	(1.8)

Values are expressed as mean and standard deviation (SD). Abbreviations: PSQI, Pittsburgh sleep quality index; BMR, basal metabolic rate; BMR_LM_, basal metabolic relativized to the lean mass; BFox, basal fat oxidation, BCHox, basal carbohydrate oxidation; MFO, maximal fat oxidation; MFO_LM_, maximal fat oxidation relativized to the lean mass; FATmax, intensity of exercise that elicits MFO; SFA, saturated fatty acids; MUFA, monounsaturated fatty acids; PUFA, polyunsaturated fatty acids; PREDIMED, PREvención con DIeta MEDiterránea.

**Table 2:** Association of sleep quality and time with BMR and BMR_LM_.

	**BMR (kcal/day)**			**BMR**_**LM**_ **(Kcal/kg**_**leanmass**_**/day)**		
	B	R^2^	P	B	R^2^	P
**PSQI global score**						
Model 0	−0.178	0.032	0.158	0.222	0.049	0.078
Model 1	−0.006	0.598	0.943	0.182	0.081	0.155
Model 2	0.026	0.616	0.760	0.143	0.107	0.269
Model 3	0.019	0.625	0.826	0.117	0.212	0.339
**Total sleep time (min)**						
Model 0	−0.459	0.211	<0.001	0.035	0.001	0.775
Model 1	−0.136	0.629	0.109	−0.079	0.053	0.557
Model 2	−0.132	0.641	0.118	−0.088	0.102	0.507
Model 3	−0.135	0.657	0.105	−0.095	0.216	0.444
**Sleep efficiency (%)**						
Model 0	−0.077	0.006	0.525	−0.018	0.000	0.879
Model 1	−0.063	0.622	0.408	−0.023	0.048	0.850
Model 2	−0.077	0.006	0.528	−0.019	0.094	0.874
Model 3	−0.074	0.007	0.550	−0.054	0.211	0.630
**Wake after sleep onset (min)**						
Model 0	0.219	0.048	0.071	−0.047	0.002	0.700
Model 1	0.113	0.627	0.141	0.079	0.054	0.516
Model 2	0.103	0.638	0.177	0.101	0.105	0.401
Model 3	0.126	0.658	0.098	0.159	0.233	0.163

The analyses were controlled for: Sex (Model 1); both sex and age (Model 2); sex, age and fat mass index (FMI) (Model 3). B, standardized linear regression coefficient; R^2^, coefficient of determination, and P value were obtained from the linear regression analyses. Bold values are values that are significant (P < 0.05). Abbreviations: PSQI, Pittsburgh sleep quality index; BMR, basal metabolic rate; BMR_LM_, basal metabolic rate relativized to the lean mass.

An inverse association was detected between PSQI global score with BFox (expressed in g/min, and as %BMR) (all P < 0.001; Table 3), which remained significant after including sex, age and FMI in the model (all P < 0.002; Table 3). We did not find any significant association between any objective sleep parameter with BFox (all P > 0.265; Table 3). PSQI was also positively associated with BCHox (expressed in g/min, and as %BMR) even after controlling for sex, age and FMI (all P < 0.002; Table S1).

**Table 3:** Association of sleep time and quality with BFox (both expressed in g/min and in %BMR).

	**BFox (g/min)**			**BFox (% BMR)**		
	B	R^2^	P	B	R^2^	P
**PSQI global score**						
Model 0	−0.475	0.225	<0.001	−0.480	0.230	<0.001
Model 1	−0.426	0.271	<0.001	−0.494	0.234	<0.001
Model 2	−0,345	0.391	0.002	−0.417	0.342	<0.001
Model 3	−0.345	0.391	0.002	−0.417	0.342	<0.001
**Total sleep time (min)**						
Model 0	−0.047	0.002	0.699	0.092	0.008	0.453
Model 1	0.100	0.089	0.450	0.122	0.012	0.375
Model 2	0.117	0.281	0.322	0.140	0.199	0.265
Model 3	0.118	0.282	0.325	0.140	0.200	0.266
**Sleep efficiency (%)**						
Model 0	0.012	0.000	0.919	0.044	0.002	0.718
Model 1	0.085	0.018	0.879	0.044	0.002	0.718
Model 2	0.010	0.274	0.924	0.037	0.150	0.743
Model 3	0.011	0.274	0.918	0.040	0.188	0,723
**Wake after sleep onset (min)**						
Model 0	0.099	0.010	0.417	0.044	0.002	0.717
Model 1	−0.061	0.085	0.608	0.043	0.002	0.727
Model 2	0.020	0.271	0.853	0.003	0.184	0.981
Model 3	0.019	0.271	0.861	−0.002	0.185	0.985

The analyses were controlled for: Sex (Model 1); both sex and age (Model 2); sex, age and fat mass index (FMI) (Model 3). B, standardized linear regression coefficient; R^2^, coefficient of determination, and P value were obtained from the linear regression analyses. Bold values are values that are significant (P < 0.05). Abbreviations: PSQI, Pittsburgh sleep quality index; BFox, basal fat oxidation (both expressed in g/min and in %BMR).

We showed an inverse association between total sleep time with MFO (expressed in g/min; P = 0.008; Table 4), which disappeared when the model includes sex, age and FMI (all P > 0.651; Table 4). No association was found between the remaining sleep parameters with MFO and MFO_LM_ (all P > 0.171; Table 4).

**Table 4:** Association of sleep time and quality with MFO and MFO_LM_.

	**MFO (g/min)**			**MFO**_**LM**_ **(g/kg**_**leanmass**_**/day)**		
	B	R^2^	P	B	R^2^	P
**PSQI global score**						
Model 0	−0.173	0.030	0.171	0.125	0.016	0.324
Model 1	−0.036	0.390	0727	0.087	0.043	0.499
Model 2	−0.020	0.394	0.851	0.036	0.092	0.784
Model 3	−0.021	0.394	0.847	0.020	0.130	0.876
**Total sleep time (min)**						
Model 0	−0.318	0.101	0.008	0.111	0.012	0.365
Model 1	−0.051	0.386	0.637	0.025	0.042	0.852
Model 2	−0.048	0.393	0.661	0.016	0.090	0.902
Model 3	−0.049	0.399	0.651	0.011	0.147	0.932
**Sleep efficiency (%)**						
Model 0	−0.022	0.000	0.856	0.033	0.001	0.783
Model 1	−0.010	0.389	0.913	0.030	0.040	0.804
Model 2	−0.022	0.001	0.857	0.037	0.035	0.757
Model 3	−0.017	0.003	0.893	0.008	0.113	0.944
**Wake after sleep onset (min)**						
Model 0	0.136	0.018	0.266	0.010	0.000	0.935
Model 1	0.052	0.386	0.598	0.039	0.043	0.752
Model 2	0.044	0.393	0.658	0.060	0.093	0.617
Model 3	0.057	0.400	0.570	0.101	0.157	0.393

The analyses were controlled for: Sex (Model 1); both sex and age (Model 2); sex, age and fat mass index (FMI) (Model 3). B, standardized linear regression coefficient; R^2^, coefficient of determination, and P value were obtained from the linear regression analyses. Bold values are values that are significant (P < 0.05). Abbreviations: PSQI, Pittsburgh sleep quality index; MFO, maximal fat oxidation; MFO_LM_, maximal fat oxidation relativized to the lean mass.

We repeated all previous associations controlling for menopausal status (pre- or post-menopausal) in order to avoid the possible cofounder of female hormones, and the results did not change (data not shown).

We observed only a negative association of fiber intake and PSQI global score and cholesterol intake negatively and positively associated with total sleep time and wake after sleep onset respectively (Table S5). However, we observed a modification effect of different dietary factors (i.e. fiber and ethanol intake; Table S5). Despite this modification effect of dietary factors and the several associations between dietary factors and sleep parameters, we did not find a mediating effect of energy, dietary energy density, fat, protein, carbohydrate, fiber intake, lipid profile intake, ethanol intake and PREDIMED total score on the association of the PSQI global score and BFox both expressed in g/min and in %BMR (Figs. S1–S4).

## Discussion

The main finding of the present study is that a poor subjective sleep quality was associated with lower BFox independently of sex, age and body composition outcomes. No consistent association was observed between any sleep quality and time parameters with BMR, MFO and FATmax. Moreover, our results indicated that the association of PSQI global score with BFox was not mediated by dietary intake and MedDiet adherence.

We observed an inverse association between total sleep time and BMR which disappeared after controlling for confounders. The energy expenditure is lowest during sleep, therefore a high total sleep time is related with a prolonged period of the lowest energy expenditure^48^. Sleep deprivation could increase energy expenditure since energy expenditure is reduced during sleep^48^. Sharma *et al*. proposed that these reduction in energy expenditure could be influenced by circadian rhythm, body temperature and muscle temperature^48^. However, the results should be interpreted with caution because this association disappeared after controlling for sex, age and FMI. Several physiological mechanisms could explain the relationship between sleep quality and BFox. Sleep restriction is associated with insulin resistance characterized by a decreased insulin-mediated glucose uptake^49^, which could develop metabolic inflexibility characterized by an impaired BFox^50^. Short sleep duration and sleep fragmentation arealso related to low leptin levels or leptin resistance^51^ which are associated with an impaired fatty acid oxidation^52^. Sleep disruption (discontinuity of sleep) can lead to the disruption of circadian rhythms^13^, which orchestrate crucial physiological and behavioral functions, being one of these the regulation of carbohydrate and fatty acid metabolism^12^. Higher sleep duration and quality are associated with a healthier gut microbiome^53^, which could suppress insulin signaling, increase β-oxidation and inhibit fat oxidation derived from the production of short-chain fatty acids^54^. Furthermore, sleep disruption (discontinuity of sleep) could decrease melatonin production^13^, which has important metabolic functions, such as lipolysis, regulating the energy flow^55^. An increase in the production of pro-inflammatory cytokines and reactive oxygen species is observed in impaired sleep patterns^13^. Both inflammation and oxidative stress could modulate metabolic flexibility, specifically fat oxidation^56,57^. Therefore, based on the above-mentioned mechanisms, a healthy sleep pattern could improve metabolic health via the increment of BFox and viceversa.

In addition, an impaired sleep pattern, determined by a low sleep duration could increase energy intake through several potential mechanisms: increment of time and opportunities for eating, psychological distress, sensitivity to food reward, energy needed to sustain wakefulness, hunger hormones and decrease dietary restraint^16^. A lack of sleep or low sleep quality could increase the intake of high energy-dense foods, high fat and sugary snacks, which are low in fiber^16^. In this sense, although we did not find any association between energy and macronutrient intake, we observed that fiber intake was negatively associated with PSQI global score. Fiber intake could have different metabolic effects (i.e. insulin sensitivity and glycemia improvement)^58^, that could have a potential role in the regulation of fat oxidation. However, we did not find any mediating role of dietary intake (i.e. fiber intake) between the association of PSQI with BFox. The lack of a mediating role may be due to specific issues: (i) since dietary outcomes were assessed in a specific time point, it could be that the dietary intake was insufficiently maintained over time to modify BFox; (ii) the possible lower and upper threshold for when dietary intake (i.e. fat intake) could modify fat oxidation^59^; (iii) the inter-individual variability, body composition and metabolic status influence on fat oxidation^8^; (iv) a sleep patterns insufficiently maintained over time.

The lack of association between any sleep outcomes with BMR and MFO could be explained by different factors. Sleep is a complex phenomenon influenced by behavioral and physiological mechanisms (i.e. homeostatic, circadian and metabolic control) under the participant’s natural sleep environment that we have not investigated^60^. These factors could influence the relationship between sleep parameters and BMR and MFO.

We also observed an inverse association between total sleep time with MFO. A previous study of Konishi *et al*. observed that a night of sleep deprivation did not affect MFO in healthy young men^15^. It has been reported several detrimental effects of long sleep for optimal health^61^. In addition, long sleep could increase fatigue, physiological deprivation, which could influence insulin resistance and hormonal imbalance^62^. Although the mechanisms are not clear, the above-mentioned mechanisms could have influenced this relationship. However, the results should be interpreted with caution because this association disappeared after controlling for sex, age and FMI.

Surprisingly, different results were observed when the association between sleep quality and energy metabolism was performed considering subjective instead of objective measures of sleep quality. It has been previously reported that PSQI and accelerometer records measure different attributes of sleep, highlighting the bias of accelerometry to register wakefulness, thus lying in bed awake but motionless is likely to be coded as sleep^63^. Therefore, it is recommended to use both methods to obtain complementary information additionally to the body movements^64^. These differences in measurement of sleep attributes could explain the different results of the associations between sleep quality and energy metabolism.

Despite accelerometer records and subjective measurements are a valid and extensively used measure of sleep quality^26,65^ they cannot differentiate between rapid eye movement sleep (REM) and non-rapid eye movement sleep (NREM), restricting the detailed assessment of the real biologic process of sleep. REM and NREM phases are metabolically different^66^. In REM sleep glucose uptake is increased, leading to anaerobic glucose metabolism^67,68^, therefore sleep quality in each phase could be differently associated with energy metabolism. Future studies that examine the relationship between REM and NREM sleep using polysomnography records with BMR and fuel oxidation in basal conditions and during exercise are needed.

The present study should be interpreted with caution; the study has a cross-sectional design that does not allow to establish causal relationship. Therefore, experimental studies should manipulate BMR and fuel oxidation and/or sleep (e.g. sleep deprivation) under well-controlled lab conditions in order to establish causal relationship. Furthermore, sleep and dietary parameters were assessed only in a specific timepoint, which do not allow us to extrapolate our results to chronic sleep or dietary patterns. Our study only included middle-aged sedentary adults and, consequently, we cannot extrapolate our results to older, younger, and/or physically active individuals. The difficulty of an accurate dietary evaluation with possible underreporting or misclassification should be considered, as in all cross-sectional studies. Lastly, the narrow PSQI global score range should be taken into account when interpreting our results.

## Conclusions

In conclusion, our study showed that a subjective poor sleep quality was associated with lower BFox. No association was found between the remaining sleep parameters with BMR and fuel oxidation in basal conditions and during exercise. Moreover, our findings indicated that the association of PSQI global score with BFox was not mediated by dietary intake and MedDiet adherence. Further studies are needed to better understand the physiological mechanisms of sleep regulation and how it could influence the BMR and fuel oxidation in basal conditions and during exercise.

## Supplementary information

Supplementary material.
