# Supplementary material for: Association between sleep quality and time with energy metabolism in sedentary adults
Source: Sci Rep. 2020 Mar 12;10:4598. doi: 10.1038/s41598-020-61493-2 (PMC7067839; doi:10.1038/s41598-020-61493-2)
Supplement: Supplementary file 1 — Supplementary material. [file 41598_2020_61493_MOESM1_ESM.docx]

Association between sleep quality and time with energy metabolism in sedentary adults

Lucas Jurado-Fasoli^1,2*^, Sol Mochón-Benguigui^1^; Manuel J. Castillo^1§^ and Francisco J. Amaro-Gahete^1,2*§^

^1^ Department of Medical Physiology. School of Medicine. University of Granada.

18016, Granada, Spain.

^2^ PROmoting FITness and Health through physical activity research group (PROFITH), Department of Physical Education and Sports, Faculty of Sport Sciences, University of Granada. Spain.

***Corresponding author**: Lucas Jurado-Fasoli & Francisco J. Amaro-Gahete. Mailing address: Department of Medical Physiology (University of Granada, Granada, Spain), Av. Investigation 11, Granada // E.mail: juradofasoli@ugr.es and amarof@ugr.es // Phone: +34 958243540 // Fax: +34 958246179 // ORCID: 0000-0002-5254-1816

**^§^** Both authors contributed equally to this work

**Acknowledgments:** The authors would like to thank all the participants that took part of the study for their time and effort. This study is part of a Ph.D. Thesis conducted in the Biomedicine Doctoral Studies of the University of Granada, Spain. We are grateful to Dr. Ángel Gutiérrez and Alejandro De la O for all their support in the study. We are grateful to Ms. Ana Yara Postigo-Fuentes for her assistance with the English language.

**SUPPLEMENTARY MATERIAL**

**Table S1.** Association of sleep time and quality with BCHox (both expressed in g/min and in %BMR).

|  | BCHox (g/min) | | | BCHox (% BMR) | | |
| --- | --- | --- | --- | --- | --- | --- |
|  | B | R^2^ | P | B | R^2^ | P |
| PSQI global score |  |  |  |  |  |  |
| Model 0 | 0.003 | 0.138 | **0.002** | 0.458 | 0.210 | **<0.001** |
| Model 1 | 0.439 | 0.224 | **0.001** | 0.485 | 0.224 | **<0.001** |
| Model 2 | 0.374 | 0.301 | **0.002** | 0.407 | 0.335 | **0.001** |
| Model 3 | 0.370 | 0.303 | **0.002** | 0.406 | 0.335 | **0.001** |
| Total sleep time (min) |  |  |  |  |  |  |
| Model 0 | -0.268 | 0.072 | **0.026** | -0.133 | 0.018 | 0.275 |
| Model 1 | -0.197 | 0.092 | 0.138 | -0.136 | 0.018 | 0.323 |
| Model 2 | -0.212 | 0.235 | 0.085 | -0.153 | 0.206 | 0.220 |
| Model 3 | -0.214 | 0.244 | 0.083 | -0.154 | 0.209 | 0.220 |
| Sleep efficiency (%) |  |  |  |  |  |  |
| Model 0 | -0.071 | 0.005 | 0.561 | -0.051 | 0.003 | 0.677 |
| Model 1 | -0.066 | 0.064 | 0.578 | -0.050 | 0.005 | 0.684 |
| Model 2 | -0.059 | 0.202 | 0.591 | -0.042 | 0.191 | 0.705 |
| Model 3 | -0.070 | 0.212 | 0.532 | -0.047 | 0.193 | 0.674 |
| Wake after sleep onset (min) |  |  |  |  |  |  |
| Model 0 | 0.002 | 0.049 | 0.687 | -0.038 | 0.001 | 0.759 |
| Model 1 | 0.016 | 0.061 | 0.896 | -0.046 | 0.005 | 0.712 |
| Model 2 | 0.052 | 0.201 | 0.648 | -0.005 | 0.188 | 0.964 |
| Model 3 | 0.068 | 0.212 | 0.552 | 0.002 | 0.190 | 0.987 |

The analyses were controlled for: Sex (Model 1); both sex and age (Model 2); sex, age and fat mass index (FMI) (Model 3). B, standardized linear regression coefficient; R^2^, and P value were obtained from the linear regression analyses. Bold values are values that are significant (P<0.05). Abbreviations: PSQI, Pittsburgh sleep quality index; BCHox, basal carbohydrate oxidation.

**Table S2.** Association of sleep time and quality with FAT_max_.

|  | FAT_max_ (% VO_2max_) | | |
| --- | --- | --- | --- |
|  | B | R^2^ | P |
| PSQI global score |  |  |  |
| Model 0 | 0.282 | 0.080 | **0.024** |
| Model 1 | 0.267 | 0.085 | **0.038** |
| Model 2 | 0.206 | 0.150 | 0.104 |
| Model 3 | 0.230 | 0.238 | 0.060 |
| Total sleep time (min) |  |  |  |
| Model 0 | 0.174 | 0.030 | 0.153 |
| Model 1 | 0.145 | 0.034 | 0.288 |
| Model 2 | 0.132 | 0.133 | 0.310 |
| Model 3 | 0.138 | 0.192 | 0.277 |
| Sleep efficiency (%) |  |  |  |
| Model 0 | -0.041 | 0.002 | 0.735 |
| Model 1 | -0.044 | 0.020 | 0.719 |
| Model 2 | -0.035 | 0.089 | 0.765 |
| Model 3 | -0.014 | 0.129 | 0.901 |
| Wake after sleep onset (min) |  |  |  |
| Model 0 | 0.022 | 0.000 | 0.859 |
| Model 1 | 0.040 | 0.018 | 0.744 |
| Model 2 | 0.071 | 0.124 | 0.547 |
| Model 3 | 0.033 | 0.178 | 0.777 |

The analyses were controlled for: Sex (Model 1); both sex and age (Model 2); sex, age and fat mass index (FMI) (Model 3). B, standardized linear regression coefficient; R^2^, and P value were obtained from the linear regression analyses. Bold values are values that are significant (P<0.05). Abbreviations: PSQI, Pittsburgh sleep quality index; FAT_max_, intensity of exercise that elicits MFO.

**Table S3.** Association of PSQI components scores with BMR, BFox, and BCHox.

|  | BMR (kcal/day) | | | BMR_LM_  (Kcal/kg_leanmass_/day) | | | BFox (g/min) | | | BFox (% BMR) | | | BCHox (g/min) | | | BCHox (% BMR) | | |
| --- | --- | --- | --- | --- | --- | --- | --- | --- | --- | --- | --- | --- | --- | --- | --- | --- | --- | --- |
|  | B | R^2^ | P | B | R^2^ | P | B | R^2^ | P | B | R^2^ | P | B | R^2^ | P | B | R^2^ | P |
| Subjective sleep quality (Component 1) |  |  |  |  |  |  |  |  |  |  |  |  |  |  |  |  |  |  |
| Model 0 | -0.216 | 0.047 | 0.087 | 0.265 | 0.070 | **0.034** | -0.361 | 0.130 | **0.003** | -0.351 | 0.123 | **0.004** | 0.235 | 0.055 | 0.061 | 0.339 | 0.115 | **0.006** |
| Model 1 | 0.048 | 0.600 | 0.580 | 0.215 | 0.090 | 0.102 | -0.288 | 0.172 | **0.023** | -0.378 | 0.129 | **0.004** | 0.341 | 0.144 | **0.009** | 0.388 | 0.134 | **0.003** |
| Model 2 | 0.076 | 0.621 | 0.382 | 0.183 | 0.118 | 0.166 | -0.213 | 0.322 | 0.068 | -0.307 | 0.266 | **0.013** | 0.281 | 0.242 | **0.024** | 0.316 | 0.271 | **0.010** |
| Model 3 | 0.057 | 0.627 | 0.519 | 0.112 | 0.210 | 0.384 | -0.220 | 0.323 | 0.068 | -0.313 | 0.267 | **0.014** | 0.276 | 0.242 | **0.032** | 0.321 | 0.272 | **0.011** |
| Sleep latency (Component 2) |  |  |  |  |  |  |  |  |  |  |  |  |  |  |  |  |  |  |
| Model 0 | -0.033 | 0.001 | 0.795 | 0.203 | 0.041 | 0.107 | -0.391 | 0.153 | **0.001** | -0.456 | 0.208 | **<0.001** | 0.390 | 0.152 | **0.001** | 0.454 | 0.206 | **<0.001** |
| Model 1 | 0.007 | 0.598 | 0.934 | 0.192 | 0.086 | 0.122 | -0.376 | 0.239 | **0.001** | -0.455 | 0.209 | **<0.001** | 0.402 | 0.202 | **0.001** | 0.456 | 0.207 | **<0.001** |
| Model 2 | 0.048 | 0.618 | 0.567 | 0.148 | 0.109 | 0.248 | -0.276 | 0.354 | **0.013** | -0.364 | 0.307 | **0.002** | 0.325 | 0.271 | **0.006** | 0.363 | 0.307 | **0.002** |
| Model 3 | 0.031 | 0.625 | 0.719 | 0.084 | 0.206 | 0.497 | -0.284 | 0.355 | **0.013** | -0.371 | 0.308 | **0.002** | 0.321 | 0.271 | **0.009** | 0.369 | 0.307 | **0.002** |
| Sleep duration (Component 3) |  |  |  |  |  |  |  |  |  |  |  |  |  |  |  |  |  |  |
| Model 0 | -0.060 | 0.004 | 0.636 | -0.023 | 0.001 | 0.860 | -0.309 | 0.096 | **0.013** | -0.323 | 0.105 | **0.009** | 0.252 | 0.063 | **0.045** | 0.292 | 0.086 | **0.019** |
| Model 1 | -0.044 | 0.600 | 0.591 | -0.027 | 0.050 | 0.828 | -0.303 | 0.190 | **0.011** | -0.323 | 0.106 | **0.010** | 0.256 | 0.106 | **0.038** | 0.293 | 0.086 | **0.020** |
| Model 2 | -0.036 | 0.617 | 0.657 | -0.040 | 0.090 | 0.749 | -0.278 | 0.360 | **0.009** | -0.297 | 0.273 | **0.009** | 0.235 | 0.229 | **0.043** | 0.267 | 0.257 | **0.020** |
| Model 3 | -0.037 | 0.626 | 0.641 | -0.046 | 0.202 | 0.695 | -0.277 | 0.360 | **0.010** | -0.297 | 0.274 | **0.010** | 0.233 | 0.234 | **0.046** | 0.267 | 0.258 | **0.021** |
| Sleep efficiency (Component 4) |  |  |  |  |  |  |  |  |  |  |  |  |  |  |  |  |  |  |
| Model 0 | -0.245 | 0.060 | 0.051 | 0.072 | 0.005 | 0.570 | -0.337 | 0.114 | **0.006** | -0.293 | 0.086 | **0.019** | 0.165 | 0.027 | 0.194 | 0.251 | 0.063 | **0.046** |
| Model 1 | -0.035 | 0.599 | 0.679 | 0.012 | 0.049 | 0.925 | -0.271 | 0.166 | **0.029** | -0.303 | 0.087 | **0.020** | 0.238 | 0.093 | 0.065 | 0.276 | 0.071 | **0.036** |
| Model 2 | -0.022 | 0.616 | 0.797 | -0.008 | 0.089 | 0.949 | -0.229 | 0.332 | **0.042** | -0.262 | 0.248 | **0.029** | 0.203 | 0.212 | 0.096 | 0.234 | 0.235 | 0.052 |
| Model 3 | -0.011 | 0.624 | 0.896 | 0.031 | 0.201 | 0.802 | -0.234 | 0.333 | **0.041** | -0.269 | 0.251 | **0.027** | 0.214 | 0.222 | 0.081 | 0.242 | 0.240 | **0.047** |
| Sleep disturbances (Component 5) |  |  |  |  |  |  |  |  |  |  |  |  |  |  |  |  |  |  |
| Model 0 | -0.230 | 0.053 | 0.068 | 0.067 | 0.005 | 0.597 | -0.374 | 0.140 | **0.002** | -0.354 | 0.125 | **0.004** | 0.240 | 0.058 | 0.056 | 0.343 | 0.118 | **0.005** |
| Model 1 | -0.072 | 0.603 | 0.385 | 0.022 | 0.050 | 0.863 | -0.323 | 0.198 | **0.008** | -0.360 | 0.126 | **0.005** | 0.295 | 0.124 | **0.019** | 0.362 | 0.126 | **0.004** |
| Model 2 | -0.013 | 0.616 | 0.889 | -0.088 | 0.095 | 0.533 | -0.155 | 0.302 | 0.215 | -0.203 | 0.216 | 0.127 | 0.158 | 0.193 | 0.240 | 0.204 | 0.217 | 0.124 |
| Model 3 | -0.004 | 0.624 | 0.968 | -0.056 | 0.202 | 0.680 | -0.158 | 0.302 | 0.213 | -0.208 | 0.219 | 0.122 | 0.167 | 0.201 | 0.219 | 0.210 | 0.221 | 0.118 |
| Use sleeping medications (Component 6) |  |  |  |  |  |  |  |  |  |  |  |  |  |  |  |  |  |  |
| Model 0 | -0.095 | 0.009 | 0.456 | 0.339 | 0.115 | **0.006** | -0.249 | 0.062 | **0.048** | -0.224 | 0.050 | 0.076 | 0.220 | 0.048 | 0.081 | 0.222 | 0.049 | 0.078 |
| Model 1 | 0.026 | 0.599 | 0.749 | 0.311 | 0.144 | **0.012** | -0.205 | 0.139 | 0.094 | -0.222 | 0.050 | 0.084 | 0.258 | 0.106 | **0.040** | 0.230 | 0.052 | 0.074 |
| Model 2 | 0.033 | 0.617 | 0.682 | 0.302 | 0.177 | **0.014** | -0.183 | 0.316 | 0.096 | -0.200 | 0.224 | 0.087 | 0.240 | 0.230 | **0.041** | 0.208 | 0.228 | 0.075 |
| Model 3 | 0.032 | 0.625 | 0.691 | 0.298 | 0.286 | **0.010** | -0.183 | 0.316 | 0.099 | -0.200 | 0.225 | 0.091 | 0.239 | 0.236 | **0.043** | 0.208 | 0.229 | 0.078 |
| Daytime dysfunction (Component 7) |  |  |  |  |  |  |  |  |  |  |  |  |  |  |  |  |  |  |
| Model 0 | 0.056 | 0.003 | 0.662 | 0.083 | 0.007 | 0.514 | -0.226 | 0.051 | 0.073 | -0.278 | 0.077 | **0.026** | 0.283 | 0.080 | **0.024** | 0.288 | 0.083 | **0.021** |
| Model 1 | 0.025 | 0.599 | 0.762 | 0.092 | 0.058 | 0.462 | -0.239 | 0.155 | **0.047** | -0.280 | 0.080 | **0.026** | 0.275 | 0.116 | **0.026** | 0.287 | 0.083 | **0.022** |
| Model 2 | 0.030 | 0.617 | 0.710 | 0.084 | 0.096 | 0.495 | -0.222 | 0.332 | **0.040** | -0.263 | 0.254 | **0.022** | 0.261 | 0.242 | **0.024** | 0.271 | 0.259 | **0.018** |
| Model 3 | 0.023 | 0.625 | 0.773 | 0.060 | 0.203 | 0.609 | -0.222 | 0.332 | **0.042** | -0.262 | 0.254 | **0.024** | 0.257 | 0.245 | **0.028** | 0.269 | 0.259 | **0.020** |

The analyses were controlled for: Sex (Model 1); both sex and age (Model 2); sex, age and fat mass index (FMI) (Model 3). B—standardized linear regression coefficient; R2, and p value were obtained from the linear regression analyses. Bold values are values that are significant (p < 0.05). Abbreviations: PSQI, Pittsburgh sleep quality index; BMR, basal metabolic rate; BFox, Basal fat oxidation; BCHox, basal carbohydrate oxidation.

**Table S4.** Association of PSQI components scores with MFO and FATmax.

|  | MFO (g/min) | | | MFO_LM_  (g/kg_leanmass_/day) | | | FAT_max_ (% VO_2max_) | | |
| --- | --- | --- | --- | --- | --- | --- | --- | --- | --- |
|  | B | R^2^ | P | B | R^2^ | P | B | R^2^ | P |
| Subjective sleep quality (Component 1) |  |  |  |  |  |  |  |  |  |
| Model 0 | -0.192 | 0.037 | 0.128 | 0.154 | 0.024 | 0.223 | 0.305 | 0.093 | **0.014** |
| Model 1 | 0.018 | 0.389 | 0.865 | 0.103 | 0.045 | 0.442 | 0.294 | 0.094 | **0.027** |
| Model 2 | 0.033 | 0.395 | 0.760 | 0.060 | 0.094 | 0.651 | 0.243 | 0.163 | 0.061 |
| Model 3 | 0.033 | 0.395 | 0.769 | 0.015 | 0.130 | 0.909 | 0.322 | 0.276 | **0.011** |
| Sleep latency (Component 2) |  |  |  |  |  |  |  |  |  |
| Model 0 | -0.057 | 0.003 | 0.653 | 0.109 | 0.012 | 0.390 | 0.213 | 0.045 | 0.092 |
| Model 1 | -0.025 | 0.389 | 0.801 | 0.100 | 0.046 | 0.428 | 0.206 | 0.059 | 0.102 |
| Model 2 | -0.005 | 0.394 | 0.963 | 0.037 | 0.092 | 0.773 | 0.130 | 0.127 | 0.305 |
| Model 3 | -0.007 | 0.394 | 0.950 | -0.003 | 0.130 | 0.982 | 0.194 | 0.224 | 0.118 |
| Sleep duration (Component 3) |  |  |  |  |  |  |  |  |  |
| Model 0 | 0.045 | 0.002 | 0.726 | 0.099 | 0.010 | 0.438 | -0.025 | 0.001 | 0.846 |
| Model 1 | 0.058 | 0.392 | 0.564 | 0.095 | 0.045 | 0.453 | -0.027 | 0.018 | 0.829 |
| Model 2 | 0.063 | 0.398 | 0.534 | 0.081 | 0.097 | 0.515 | -0.047 | 0.114 | 0.703 |
| Model 3 | 0.063 | 0.398 | 0.538 | 0.077 | 0.136 | 0.529 | -0.041 | 0.192 | 0.725 |
| Sleep efficiency (Component 4) |  |  |  |  |  |  |  |  |  |
| Model 0 | -0.193 | 0.037 | 0.127 | 0.054 | 0.003 | 0.669 | 0.239 | 0.057 | 0.057 |
| Model 1 | -0.023 | 0.389 | 0.825 | 0.003 | 0.036 | 0.985 | 0.220 | 0.062 | 0.093 |
| Model 2 | -0.016 | 0.394 | 0.883 | -0.022 | 0.091 | 0.866 | 0.190 | 0.145 | 0.132 |
| Model 3 | -0.015 | 0.394 | 0.890 | 0.001 | 0.130 | 0.991 | 0.160 | 0.214 | 0.193 |
| Sleep disturbances (Component 5) |  |  |  |  |  |  |  |  |  |
| Model 0 | -0.204 | 0.042 | 0.106 | 0.042 | 0.002 | 0.740 | 0.337 | 0.113 | **0.007** |
| Model 1 | -0.078 | 0.394 | 0.447 | 0.003 | 0.036 | 0.981 | 0.324 | 0.117 | **0.011** |
| Model 2 | -0.055 | 0.396 | 0.635 | -0.133 | 0.104 | 0.347 | 0.227 | 0.151 | 0.101 |
| Model 3 | -0.054 | 0.396 | 0.642 | -0.114 | 0.140 | 0.416 | 0.201 | 0.221 | 0.134 |
| Use sleeping medications (Component 6) |  |  |  |  |  |  |  |  |  |
| Model 0 | -0.189 | 0.036 | 0.135 | 0.116 | 0.013 | 0.363 | 0.215 | 0.046 | 0.088 |
| Model 1 | -0.094 | 0.397 | 0.354 | 0.088 | 0.043 | 0.489 | 0.199 | 0.056 | 0.119 |
| Model 2 | -0.090 | 0.402 | 0.376 | 0.076 | 0.096 | 0.541 | 0.184 | 0.145 | 0.134 |
| Model 3 | -0.090 | 0.402 | 0.379 | 0.074 | 0.135 | 0.548 | 0.187 | 0.225 | 0.113 |
| Daytime dysfunction (Component 7) |  |  |  |  |  |  |  |  |  |
| Model 0 | -0.039 | 0.002 | 0.760 | -0.033 | 0.001 | 0.798 | 0.079 | 0.006 | 0.533 |
| Model 1 | -0.064 | 0.392 | 0.523 | -0.025 | 0.036 | 0.843 | 0.085 | 0.024 | 0.506 |
| Model 2 | -0.061 | 0.398 | 0.544 | -0.034 | 0.092 | 0.782 | 0.073 | 0.117 | 0.552 |
| Model 3 | -0.062 | 0.398 | 0.542 | -0.049 | 0.132 | 0.687 | 0.094 | 0.199 | 0.425 |

The analyses were controlled for: Sex (Model 1); both sex and age (Model 2); sex, age and fat mass index (FMI) (Model 3). B, standardized linear regression coefficient; R^2^, and p value were obtained from the linear regression analyses. Bold values are values that are significant (p < 0.05). Abbreviations: PSQI, Pittsburgh sleep quality index; MFO, maximal fat oxidation; MFO_LM_, maximal fat oxidation relativized to the lean mass; FAT_max_, intensity of exercise that elicits MFO.

**Table S5.** Association of dietary factors and sleep parameters.

|  | PSQI global score | | Total sleep time | | Sleep efficiency | | Wake after sleep onset | |
| --- | --- | --- | --- | --- | --- | --- | --- | --- |
|  | r | P | r | P | r | P | r | P |
| Energy (kcal) | -0.120 | 0.365 | 0.058 | 0.646 | 0.133 | 0.290 | -0.167 | 0.186 |
| Dietary energy density (kcal/g/day) | -0.165 | 0.195 | -0.053 | 0.669 | 0.020 | 0.872 | -0.006 | 0.962 |
| Fat (g/day) | -0.141 | 0.287 | -0.070 | 0.583 | 0.020 | 0.873 | -0.020 | 0.874 |
| Protein (g/day) | -0.105 | 0.430 | 0.110 | 0.388 | 0.036 | 0.774 | -0.196 | 0.120 |
| Carbohydrate (g/day) | -0.092 | 0.489 | 0.114 | 0.368 | 0.183 | 0.144 | -0.199 | 0.116 |
| Fiber (g/day) | -0.281 | **0.031** | 0.128 | 0.315 | 0.069 | 0.584 | -0.103 | 0.416 |
| Ethanol (g/day) | 0.040 | 0.762 | -0.108 | 0.396 | 0.057 | 0.651 | -0.008 | 0.947 |
| SFA (g/day) | -0.092 | 0.473 | -0.163 | 0.185 | -0.016 | 0.898 | -0.126 | 0.307 |
| MUFA (g/day) | 0.000 | 0.998 | -0.205 | 0.094 | 0.044 | 0.718 | 0.027 | 0.827 |
| PUFA (g/day) | 0.023 | 0.856 | -0.131 | 0.287 | 0.122 | 0.317 | -0.132 | 0.282 |
| Cholesterol (mg/day) | 0.109 | 0.394 | -0.326 | **0.007** | -0.163 | 0.180 | 0.367 | **0.002** |
| PREDIMED total score | 0.091 | 0.480 | 0.150 | 0.226 | 0.006 | 0.960 | -0.027 | 0.827 |

Pearson correlations were performed. Values in bold are P<0.05

**Table S6.** Effect modification analyses of dietary intake and PSQI global score on basal fat oxidation.

|  | **BFox (g/min)** | | | **BFox (% BMR)** | | |
| --- | --- | --- | --- | --- | --- | --- |
|  | B | R^2^ | P | B | R^2^ | P |
| Energy (kcal) * PSQI interaction | -0.297 | 0.088 | 0.022 | -0.327 | 0.107 | 0.011 |
| Dietary energy density (kcal/g/day) * PSQI interaction | -0.405 | 0.164 | 0.001 | -0.440 | 0.194 | <0.001 |
| Fat (g/day) * PSQI interaction | -0.330 | 0.109 | 0.008 | -0.386 | 0.149 | 0.002 |
| Protein (g/day) * PSQI interaction | -0.296 | 0.088 | 0.017 | -0.324 | 0.105 | 0.009 |
| Carbohydrate (g/day) * PSQI interaction | -0.349 | 0.122 | 0.005 | -0.385 | 0.148 | 0.002 |
| Fiber (g/day) * PSQI interaction | -0.039 | 0.002 | 0.768 | -0.061 | 0.004 | 0.646 |
| Ethanol (g/day) * PSQI interaction | -0.142 | 0.020 | 0.275 | -0.185 | 0.034 | 0.154 |
| SFA (g/day) * PSQI interaction | -0.312 | 0.097 | 0.013 | -0.390 | 0.152 | 0.002 |
| MUFA (g/day) * PSQI interaction | -0.326 | 0.106 | 0.009 | -0.378 | 0.143 | 0.002 |
| PUFA (g/day) * PSQI interaction | -0.261 | 0.068 | 0.039 | -0.295 | 0.087 | 0.019 |
| Cholesterol (mg/day) * PSQI interaction | -0.313 | 0.098 | 0.013 | -0.341 | 0.117 | 0.006 |
| PREDIMED total score* PSQI interaction | -0.469 | 0.220 | 0.000 | -0.465 | 0.216 | <0.001 |

R^2^, and p value were obtained from the linear regression analyses. Abbreviations: PSQI, Pittsburgh sleep quality index; BMR, basal metabolic rate; BFox, Basal fat oxidation.


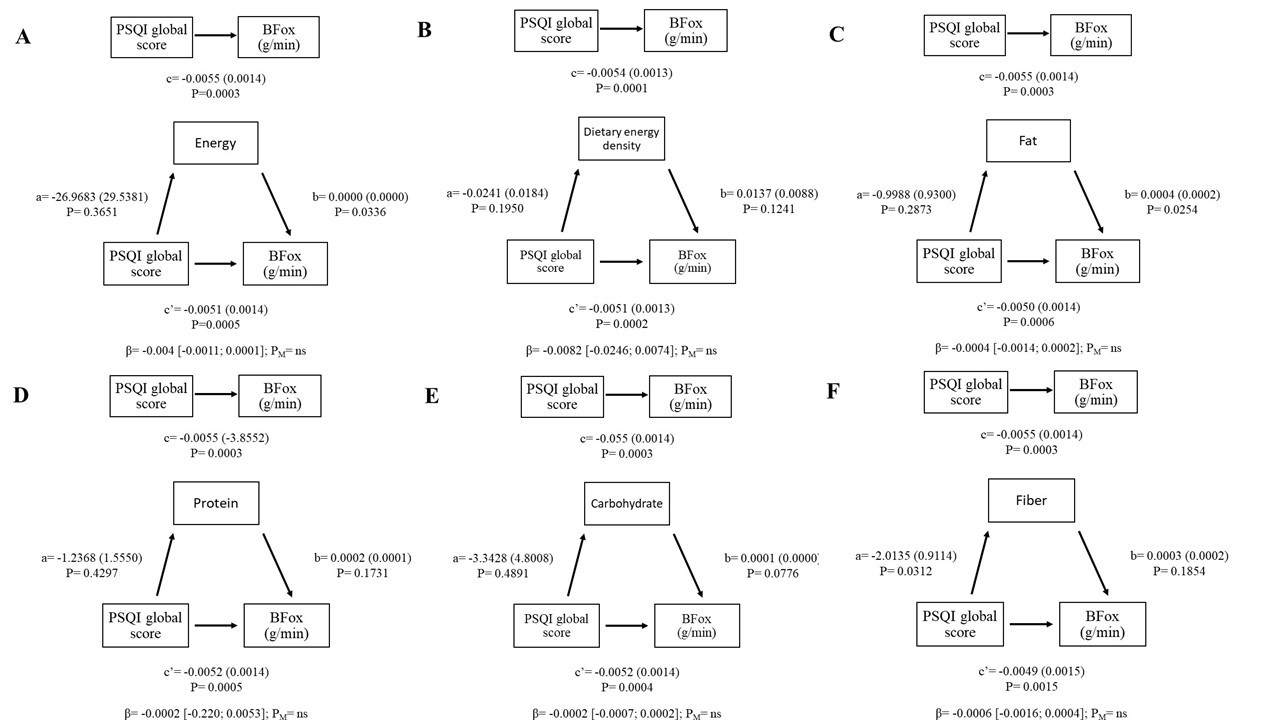


**Figure S1.** Mediation models of the relationship between PSQI global score and BFox with (A) energy intake, (B) dietary energy density intake, (C) fat intake, (D) protein intake, (E) carbohydrate intake, and (F) fiber intake included as mediator variables. Paths a, b, c and c’ are presented as unstandardized coefficients (SE). β= indirect effect (a x b paths) [lower-limit CI; upper-limit CI], lower and upper levels for bias-corrected 95% Cis of the indirect effect based on 5,0000 bootstraps. Abbreviations: PSQI, Pittsburgh sleep quality index, BFox, basal fat oxidation; CI, confidence interval; ns, nonsignificant.


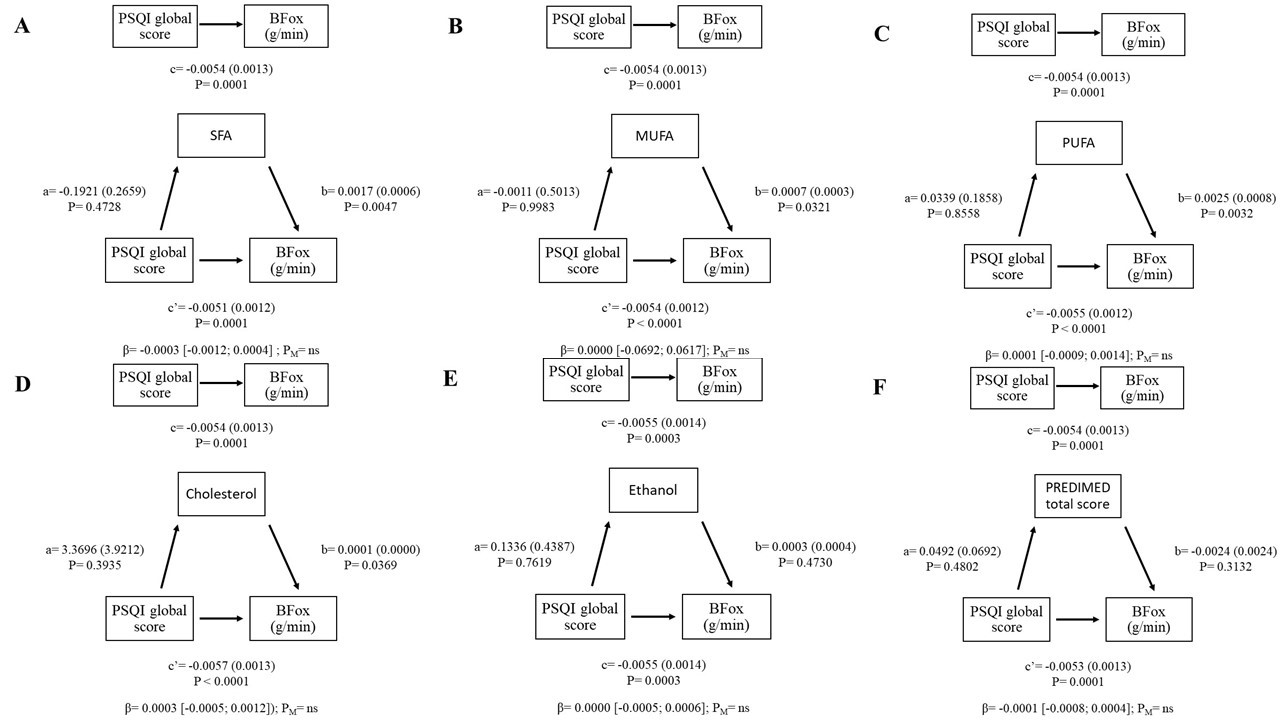


**Figure S2**. Mediation models fiber intake of the relationship between PSQI global score and BFox with (A) SFA intake, (B) MUFA intake, (C) PUFA intake, (D) cholesterol intake, (E) ethanol intake, and (F) PREDIMED total score included as mediator variables. Paths a, b, c and c’ are presented as unstandardized coefficients (SE). β= indirect effect (a x b paths) [lower-limit CI; upper-limit CI], lower and upper levels for bias-corrected 95% Cis of the indirect effect based on 5,0000 bootstraps. Abbreviations: PSQI, Pittsburgh sleep quality index, BFox, basal fat oxidation; SFA, saturated fatty acids; MUFA, monounsaturated fatty acids; PUFA, polyunsaturated fatty acids; PREDIMED, PREvención con DIeta MEDiterránea; CI, confidence interval; ns, nonsignificant.


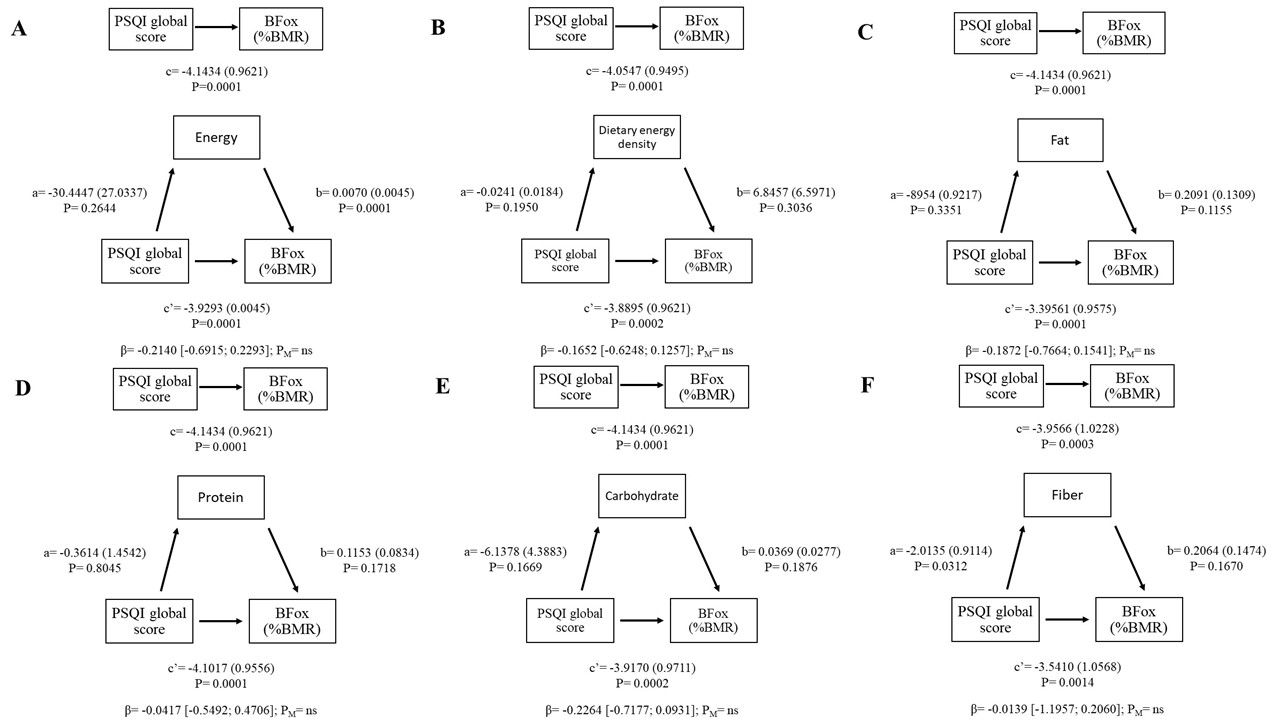


**Figure S3.** Mediation models of the relationship between PSQI global score and BFox expressed as percentage of BMR with (A) energy intake, (B) dietary energy density intake, (C) fat intake, (D) protein intake, (E) carbohydrate intake, and (F) fiber intake included as mediator variables. Paths a, b, c and c’ are presented as unstandardized coefficients (SE). β= indirect effect (a x b paths) [lower-limit CI; upper-limit CI], lower and upper levels for bias-corrected 95% Cis of the indirect effect based on 5,0000 bootstraps. Abbreviations: BMR, basal metabolic rate; PSQI, Pittsburgh sleep quality index, BFox, basal fat oxidation; CI, confidence interval; ns, nonsignificant.


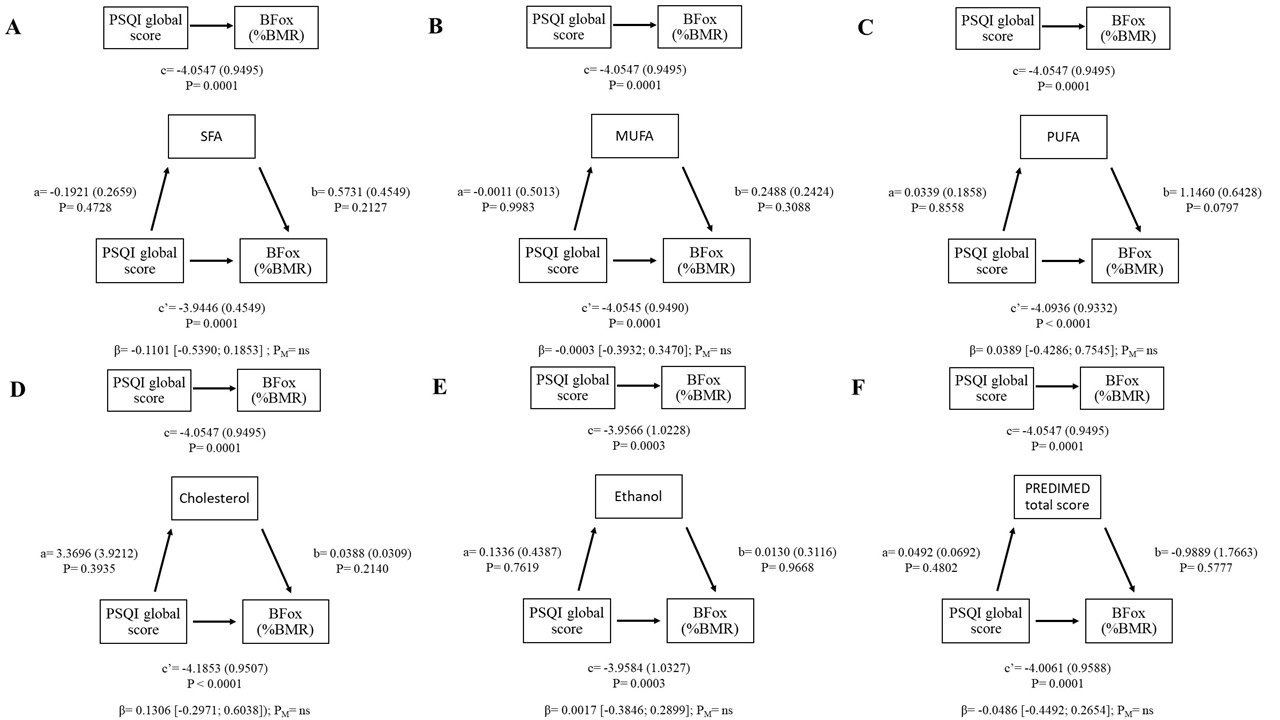


**Figure S4.** Mediation models fiber intake of the relationship between PSQI global score and BFox with (A) SFA intake, (B) MUFA intake, (C) PUFA intake, (D) cholesterol intake, (E) ethanol intake, and (F) PREDIMED total score included as mediator variables. Paths a, b, c and c’ are presented as unstandardized coefficients (SE). β= indirect effect (a x b paths) [lower-limit CI; upper-limit CI], lower and upper levels for bias-corrected 95% Cis of the indirect effect based on 5,0000 bootstraps. Abbreviations: PSQI, Pittsburgh sleep quality index, BFox, basal fat oxidation; BMR, basal metabolic rate; SFA, saturated fatty acids; MUFA, monounsaturated fatty acids; PUFA, polyunsaturated fatty acids; PREDIMED, PREvención con DIeta MEDiterránea; CI, confidence interval; ns, nonsignificant.
